# Applying Meta-Analytic Structural Equation Modeling to Examine the Relationships Among Work Stress, Job Burnout, and Turnover Intention in Taiwanese Nurses

**DOI:** 10.3390/healthcare13212718

**Published:** 2025-10-27

**Authors:** Yi-Horng Lai, Mei-Yun Chang, Chung-Cheng Wang

**Affiliations:** 1 Department of Healthcare Administration, Asia Eastern University of Science and Technology, New Taipei City 220303, Taiwan; 2 Division of Respiratory Therapy, Department of Internal Medicine, Far Eastern Memorial Hospital, New Taipei City 220, Taiwan; changmy30@yahoo.com.tw; 3 En Chu Kong Hospital, New Taipei City 237, Taiwan; ericwcc@ms27.hinet.net

**Keywords:** nurses, turnover intention (TI), work stress (WS), job burnout (JB)

## Abstract

**Background/Objectives:** Nursing staff are essential to healthcare delivery, yet Taiwan has experienced a significant rise in nurse turnover in recent years. Retention has thus become a critical concern for healthcare institutions. Identifying the factors influencing nurses’ turnover intentions (TIs) and improving workplace conditions may help to reduce attrition. This study investigates the relationships among TI, work stress (WS), and job burnout (JB), examining variations across healthcare settings and comparing the periods before and after the COVID-19 pandemic. **Methods:** This study systematically reviews 28 studies published between 2011 and 2025, retrieved from Taiwan’s Master’s and Doctoral Thesis Knowledge Value Added System, Airiti Library, and Google Scholar. The review was conducted in accordance with the Preferred Reporting Items for Systematic Reviews and Meta-Analyses Protocols (PRISMA) guidelines. The data were analyzed using a combined approach of meta-analysis and structural equation modeling. **Results:** The findings of this study indicate that WS has a statistically significant impact on TI (path coefficient = 0.281, 95% CI: 0.102 to 0.459, *p* = 0.002). Similarly, JB significantly influences TI (path coefficient = 0.342, 95% CI: 0.163 to 0.520, *p* < 0.001). WS also has a strong and significant effect on JB (path coefficient = 0.612, 95% CI: 0.485 to 0.739, *p* < 0.001). These results suggest that WS has a particularly strong effect on JB among nurses working in non-medical center hospitals in Taiwan. Additionally, no significant differences were found in the relationships among TI, WS, and JB before and after the COVID-19 pandemic. **Conclusions:** Based on the findings of this study, it is recommended that healthcare administrators closely monitor the stress experienced by nursing staff and identify the key factors that lead to WS and JB. Developing targeted policies for different healthcare settings may help to reduce nurses’ intentions to leave their jobs.

## 1. Introduction

In recent years, the high turnover rate and shortage of nursing staff have become major challenges in healthcare systems [1]. Nurses represent the largest group of healthcare professionals and are responsible for complex and demanding tasks. Their role involves providing continuous care within strict time limits, requiring constant attention and timely completion of duties. Nurses offer direct, comprehensive care that is adjusted to patients’ different health conditions and levels of illness, while also supporting the needs of families and caregivers. These responsibilities contribute to increased workload and stress. Managing such demands effectively depends heavily on clinical experience, highlighting the important role of experienced nurses in hospital operations [2]. When nurses leave their jobs, hospitals lose essential clinical skills, and nurses’ professional development is disrupted. These outcomes result in significant losses for the healthcare system [3].

Nursing involves a demanding workload that includes caring for seriously ill patients, managing death, and navigating complex communication with patients, families, and colleagues in a tense and uncertain environment [1]. These challenges make nursing a highly stressful and skilled profession. When stress becomes overwhelming, it can cause physical, psychological, and behavioral issues, leading to fatigue. This fatigue may lower work efficiency, reduce team morale, and increase turnover. Rotating shifts, though necessary for patient care, disrupt sleep patterns and contribute to exhaustion. Nurses also handle additional administrative duties and personal responsibilities, which may create conflicting demands and role stress. Poor support, unclear expectations, and ineffective task delegation can further impair performance, leading to burnout and withdrawal behaviors [4]. As a result, some nurses leave the profession, raising concerns about the impact of high turnover on healthcare quality [5].

Nursing is a helping profession that involves frequent interaction with others and requires high professional skills and emotional strength. Constant exposure to patients’ suffering can cause job burnout (JB) and emotional exhaustion. Additionally, nurses often work under time and resource constraints, which increases their stress and challenges. Therefore, protecting nurses’ physical and mental health is essential. Providing necessary support can help prevent the negative effects of burnout and emotional exhaustion on both nurses and their patients [6].

The high turnover rate among nursing personnel poses a substantial challenge for healthcare systems in Taiwan and across the globe [7]. Data from Taiwan’s Ministry of Health and Welfare indicate that nurse turnover rates were 10.13% in 2021, 11.73% in 2022, 12.61% in 2023, and 11.58% in 2024 [8]. Nurse retention remains a critical concern for hospitals, as maintaining a stable and adequately staffed nursing workforce is essential for ensuring high-quality patient care. Moreover, it directly influences the operational efficiency and public perception of healthcare institutions [9]. Elevated turnover rates can compromise healthcare facility performance, necessitating increased investment in the recruitment, selection, and training of replacement staff. This process not only incurs additional costs but also exacerbates the workload of remaining nurses, potentially prompting further resignations and perpetuating a cycle of turnover [10]. In the long term, these problems may harm the hospital’s reputation and overall performance [10].

Reducing nurse turnover is a key responsibility for hospital management [7]. Waiting until nurses submit their resignation before taking action may be too late. Therefore, if hospitals can identify the reasons behind nurses’ intentions to leave before they resign, they can make proactive changes to address these issues. This preventive approach can help to lower turnover rates. Research has shown that turnover intention is the strongest predictor of actual turnover [11]. When feeling dissatisfied, employees usually develop turnover intentions (TI), which often lead to resignation [9]. Thus, understanding and addressing the factors that cause nurses to consider leaving can help to prevent turnover. Studies have found that work stress (WS) and JB are strongly linked to TI [12]. In particular, higher levels of WS and JB increase nurses’ desire to leave their jobs.

WS refers to the psychological and physiological responses that arise when job demands exceed an individual’s available resources or capabilities. According to the World Health Organization, WS results from a discrepancy between job demands and personal competence, leading to difficulties in coping effectively [13]. Lazarus and Folkman’s Stress-Coping Model underscores that stress is based on subjective appraisal, wherein perceived inadequacy of coping resources leads to emotional and physical strain [14]. Nurses are particularly exposed to a wide range of complex stressors, including heavy workloads, patient aggression, lack of social support, role ambiguity, shift work, and academic pressures. Additional contributing factors include limited resources, time constraints, administrative burdens, and challenges in maintaining a balance between work and personal life. A substantial body of research has demonstrated a positive association between WS and increased TIs among nurses [15,16,17]. However, some research suggests that stress stemming from the work environment may not significantly influence nurses’ TI [18]. Nevertheless, WS has been shown to negatively impact job satisfaction and organizational commitment [19], both of which are critical predictors of TI.

JB was first conceptualized by Freudenberger to describe emotional exhaustion and psychological depletion among professionals working in environments characterized by sustained high levels of stress [20]. Maslach and Jackson later expanded this concept by defining JB as a chronic psychological response to stress experienced in the workplace [21]. In clinical settings, nurses are particularly vulnerable to JB due to factors such as rotating shifts, repetitive duties, limited resources, and intensive emotional demands. Care workers who are responsible for direct patient care and emotional support are similarly affected by these conditions. Extensive research has established a positive relationship between JB and increased TIs among nurses [6,22]. Although JB may not directly influence TI, it affects important contributing factors, such as organizational commitment, which play a significant role in shaping employees’ intention to leave [23].

The type of hospital where nurses work can affect how they experience work related stress. Variations in hospital characteristics, including patient volume, severity of illness, and availability of medical equipment, affect the demands placed on nursing staff. Nurses in medical centers frequently face higher expectations related to their professional expertise and patient safety responsibilities. Moreover, they often operate in more complex work environments and manage heavier caregiving workloads compared to their counterparts in district hospitals [24,25]. As a result, nurses in higher level healthcare institutions tend to have higher levels of stress [26].

During the COVID-19 pandemic, frontline nurses showed remarkable resilience while confronting the virus directly. However, the uncertainty of a rapidly changing situation and the invisible nature of the virus caused significant psychological and work-related stress [27]. Factors contributing to this stress included heavier workload due to frequent changes in health policies, shortages of protective equipment, limited early knowledge about infection prevention, staff shortages, and a steady flow of suspected and confirmed cases. Hospitals had to manage existing patients while reallocating resources to treat new COVID-19 cases. As hospital capacity was stretched, the increased risk of exposure negatively impacted nurses’ physical and mental health, job performance, and future career plans [28]. Many studies have reported a notable rise in nurses’ TIs after the pandemic [28]. Additionally, the increased demands during the crisis have been linked to higher levels of JB and WS among nurses [29].

Meta-Analytic Structural Equation Modeling (MASEM) is a method that combines meta-analysis and structural equation modeling (SEM) to examine complex relationships across multiple studies. MASEM helps to confirm uncertain research findings by combining relevant past studies and analyzing the correlations between variables. This approach enables more robust and generalizable conclusions, which can inform practical applications and guide future research [30].

This study departs from prior research, which predominantly analyzed Taiwanese nurses’ TI, WS, and JB as independent variables. It explores the interrelationships and cumulative effects of these factors through an integrated meta-analytic framework combined with structural equation modeling. The study also compares these factors across different types of healthcare institutions and examines changes before and after the COVID-19 pandemic. The findings aim to provide valuable insights for health authorities and hospital administrators in formulating effective policies and support systems to retain skilled nursing staff, reduce turnover rates, and enhance the quality and efficiency of healthcare services.

## 2. Materials and Methods

The main goal of this study is to explore the relationships between TI, WS, and JB among nurses. A meta-analysis is used to systematically gather and combine existing research data on these topics. This approach helps clarify the key aspects of each variable and allows for the analysis of how they are related. Effect sizes are also calculated to measure the strength and direction of these relationships.

Subsequently, SEM is employed to test the proposed theoretical framework and to examine the relationships among the key variables. The research model is developed based on a thorough review of relevant literature to ensure both theoretical support and practical relevance. Based on previous studies, the following research hypotheses are proposed:

**Hypothesis** **1** **(H1):** *Increased levels of WS are associated with greater TI among nurses*.

**Hypothesis** **2** **(H2):** *Elevated levels of JB are associated with greater TI among nurses*.

**Hypothesis** **3** **(H3):** *Higher levels of WS are associated with elevated levels of JB among nurses*.

**Hypothesis** **4** **(H4):** *WS influences TI indirectly through JB*.

The study was prospectively registered with the International Platform of Registered Systematic Review and Meta-analysis Protocols (INPLASY; Registration No. INPLASY2025100094, DOI: 10.37766/inplasy2025.10.0094).

### 2.1. Research Materials

This study employed a meta-analytic methodology to synthesize data and samples from previously published empirical research conducted between 2011 and 2025. The review process adhered to the Preferred Reporting Items for Systematic Reviews and Meta-Analyses (PRISMA) guidelines [31]. The PRISMA 2020 Checklist is included in Appendix A.

This review followed the updated PRISMA guidelines [31]. Studies published before 28 February 2025, were selected from the National Digital Library of Theses and Dissertations in Taiwan, Google Scholar, and Airiti Library. The search terms used were: (“turnover intention”) OR (“job stress” OR “work stress”) OR (“job burnout” OR “work burnout”). To reduce the risk of publication and source selection bias, a manual search was additionally conducted.

We included questionnaire-based behavioral science studies that collected responses from nurse samples. After initial inclusion based on the review topic, studies were excluded if they met any of the following criteria: (a) the sample did not include nurses (Criterion 1: other population); (b) no direct relationships were reported between any two of these variables: WS, JB, and TI (Criterion 2: other measures); (c) there was insufficient statistical data to calculate effect sizes (Criterion 3: lack of data); (d) the article was not published in Chinese or English (Criterion 4: inaccessible language); or (e) the publication was a review, commentary, case series, or methodological paper (Criterion 5: wrong publication type). Additionally, studies containing duplicated data were excluded. In cases where multiple publications utilized the same dataset, only the most recent study was included (Criterion 6: duplicated data).

In accordance with the guidelines proposed by Higgins and Green [32], two researchers independently and blindly screened the titles and abstracts after the removal of duplicate records. Irrelevant studies were excluded, and the remaining ones were retained. The screening was then unblinded. The researchers also reviewed the reference lists of the included empirical studies and reviews to identify additional relevant studies not found in the initial search. Both researchers read all retained studies and independently assessed their eligibility. Discrepancies arising at any stage were addressed and resolved through collaborative discussion.

Two independent reviewers evaluated the methodological quality of the included studies using the Appraisal Tool for Cross-Sectional Studies (AXIS), a validated instrument specifically designed for the critical appraisal of observational cross-sectional research [33]. The AXIS tool comprises 20 items that assess key components of study design and reporting across the introduction, methods, results, and discussion sections. Each item was rated as “yes,” “no,” or “don’t know.” Discrepancies between reviewers were resolved through consensus.

Data from each included study were systematically extracted using a data extraction sheet specifically developed for this review and updated as needed. Where applicable, the following information was extracted: year of publication, sample size, type of institution where data were obtained, geographic location of the institution, year of data collection, and correlation coefficients between TI and WS, TI and JB, and WS and JB.

**Figure 1:**
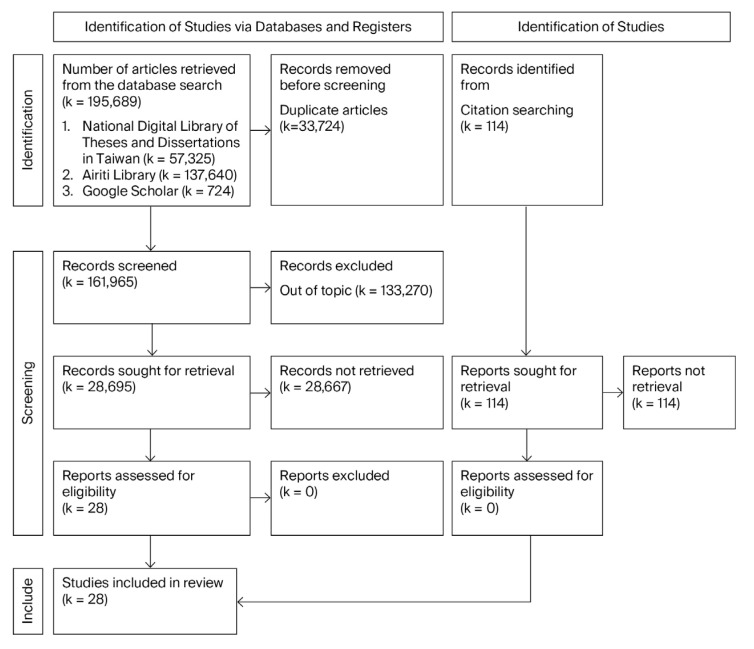
PRISMA flow diagram.

A total of 195,689 master’s and doctoral theses with accessible full texts were initially retrieved through the database search. Studies that contained incomplete data or adopted qualitative research methodologies were excluded from further analysis. After applying the inclusion and exclusion criteria, a final sample of 28 quantitative studies was selected for meta-analysis, as illustrated in Figure 1.

### 2.2. Data Analysis Methods

This study used a two-phase analytical approach. In the first phase, meta-analysis was conducted to systematically collect and combine relevant research data. In the second phase, SEM was used to test and confirm the proposed theoretical model. This step-by-step process ensured thorough data integration and careful evaluation of the model.

In the meta-analysis phase, the main goal was to systematically review existing studies and gather data related to the study variables. This helped create a comprehensive dataset for further analysis. In the SEM phase, structural equation modeling techniques were used for detailed statistical analysis. The model’s fit was assessed using the data collected during the meta-analysis phase. Path analysis, confirmatory factor analysis (CFA), and full structural equation modeling were employed to evaluate the proposed relationships among the variables

Following the estimation of correlation coefficients among WS, JB, and TI through meta-analysis, this study utilized SAS 9.3 to perform SEM for data analysis. SEM is a comprehensive statistical technique for examining causal relationships by modeling both direct and indirect effects among observed and latent variables. Particularly suited for analyzing complex constructs that cannot be directly measured, SEM integrates the strengths of factor analysis and path analysis, thereby addressing the limitations of each. Unlike traditional path analysis, which assumes error-free measurement, SEM explicitly accounts for measurement error and provides detailed model diagnostics, including fit indices and modification indices. These features enhance the precision, reliability, and validity of theoretical model testing and development.

This study used SEM to build the research model and empirically examine the relationships among key variables. Specifically, it analyzed the effects of WS and JB on nurses’ TI. The study also explored how these relationships differ across various types of healthcare institutions and examined changes before and after the COVID-19 pandemic in Taiwan, focusing on how WS and JB influence TI in different organizational and regional settings.

## 3. Results

### 3.1. Sample Data Description

This study utilized a meta-analytic approach to synthesize findings from 28 selected studies (see Table 1). Among these, 24 were master’s or doctoral theses [34,35,36,37,38,39,40,41,42,43,44,45,46,47,48,49,50,51,52,53,54,55,56,57], while the remaining 4 were peer-reviewed journal articles [12,19,58,59]. Five studies (17.86%) specifically examined nurses working in medical centers. Notably, 11 studies (39.28%) were published after 2020, aligning with the onset of the COVID-19 pandemic. Additionally, more than half of the studies (n = 17; 60.71%) included sample sizes greater than 200 participants, with an overall mean sample size of 259.29.

### 3.2. Outcomes of the Meta-Analysis

#### 3.2.1. The Relationship Between Work Stress and Turnover Intention

**Figure 2:**
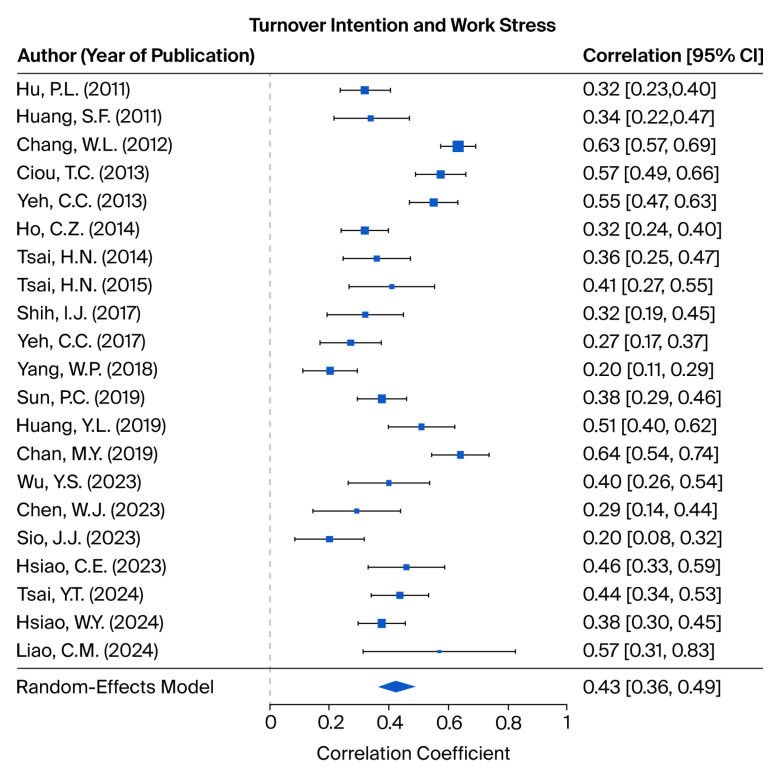
Forest plots depicting the tested hypotheses concerning the relationship between work-related stress and turnover intention, as measured by correlation coefficients [12,19,34,35,36,37,38,41,42,43,44,45,46,49,50,51,52,54,55,56,58].

This study investigated the relationship between WS and TI based on 21 research samples. The test of homogeneity produced a Q statistic of 160.7653, which was statistically significant (*p* < 0.001), thereby rejecting the null hypothesis of homogeneity and indicating substantial heterogeneity among the included studies. Consequently, a random-effects model was employed. The I^2^ statistic was 85.33%, further confirming a high level of heterogeneity. The meta-analysis produced a statistically significant effect size of 0.4263 (95% CI = 0.3634 to 0.4893), indicating a moderate positive association between WS and TI. These findings are illustrated in Figure 2. The leave-one-out sensitivity analysis is presented in Appendix C, Table A5, demonstrating that the exclusion of any individual study does not significantly impact the overall results.

**Figure A2:**
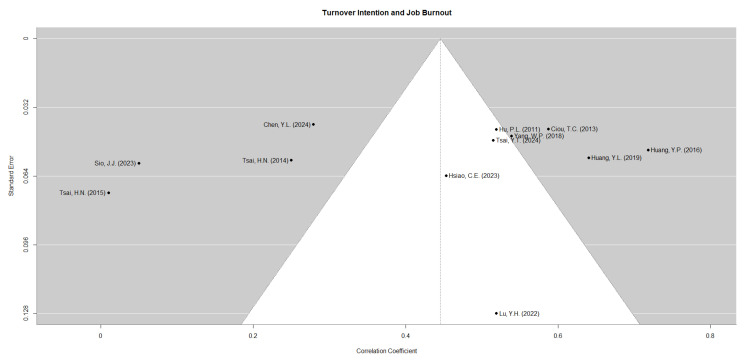
Funnel plot displaying the effect size estimates from the meta-analysis of job burnout and nurses’ turnover intention in studies published between 2011 and 2025 (k = 12) [12,34,37,38,39,43,44,48,49,52,53,54].

As part of the assessment of publication bias, the fail-safe N was calculated. The results indicated that 10,332 additional unpublished or missing studies with null results would be required to reduce the observed effect size between WS and TI to non-significance. This number substantially exceeds the calculated tolerance threshold of 110 studies, indicating that the findings of the present meta-analysis are robust and unlikely to be significantly affected by publication bias. A funnel plot illustrating the distribution of effect size estimates from the included studies is provided in Appendix B, Figure A2. Moreover, Egger’s test for funnel plot asymmetry produced non-significant results (intercept = 0.6240, *p* = 0.4815), providing additional evidence against the presence of publication bias.

#### 3.2.2. The Relationship Between Job Burnout and Turnover Intention

**Figure 3:**
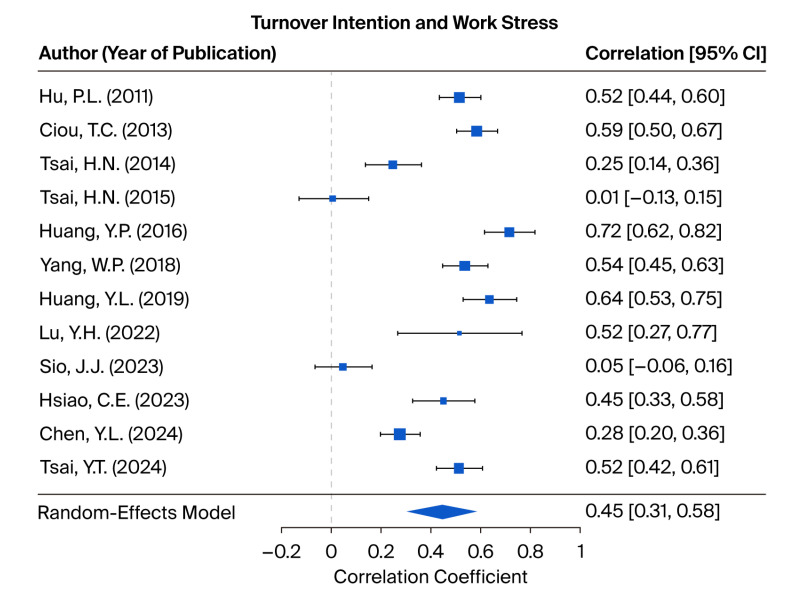
Forest plots depicting the tested hypotheses concerning the relationship between job burnout and turnover intention, as measured by correlation coefficients [12,34,37,38,39,43,44,48,49,52,53,54].

This study investigated the relationship between JB and TI based on 12 research samples. The test of homogeneity produced a Q statistic of 172.5949, which was statistically significant (*p* < 0.001), thereby rejecting the null hypothesis of homogeneity and indicating substantial heterogeneity among the included studies. Consequently, a random-effects model was employed. The I^2^ statistic was 94.56%, further confirming a high level of heterogeneity. The meta-analysis yielded a statistically significant effect size of 0.4457 (95% CI = 0.3084 to 0.5830), suggesting a moderate positive association between JB and TI. These findings are illustrated in Figure 3. The leave-one-out sensitivity analysis is presented in Appendix C, Table A6, demonstrating that the exclusion of any individual study does not significantly impact the overall results.

As part of the publication bias assessment, the fail-safe N was calculated to be 3513, indicating that this number of unpublished or missing studies with null results would be required to render the observed effect between JB and TI non-significant. This figure substantially exceeds the tolerance threshold of 70 studies, suggesting that the overall findings are robust and unlikely to be substantially affected by publication bias. A funnel plot displaying the distribution of effect size estimates from the included studies is provided in Appendix B, Figure A2. Furthermore, Egger’s test for funnel plot asymmetry was not statistically significant (intercept = 0.6240, *p* = 0.4815), offering additional evidence against the existence of publication bias.

#### 3.2.3. The Relationship Between Work Stress and Job Burnout

**Figure 4:**
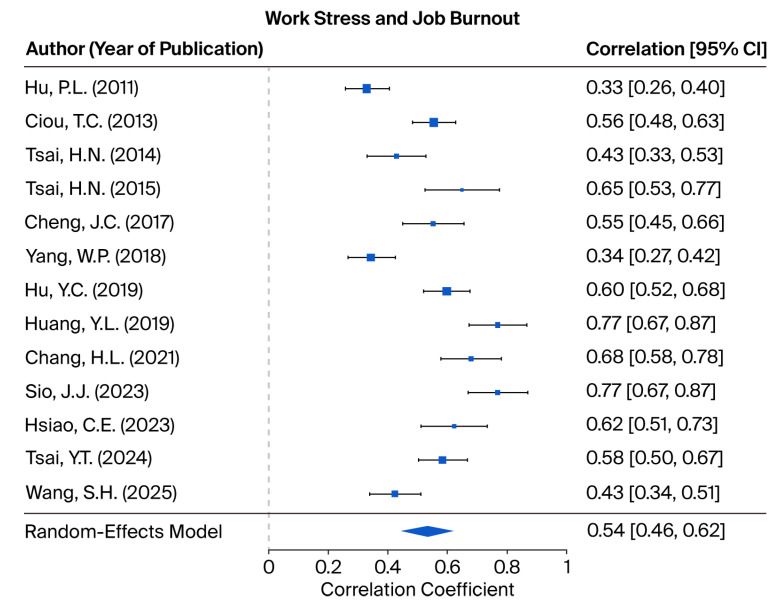
Forest plots depicting the tested hypotheses concerning the relationship between work stress and job burnout, as measured by correlation coefficients [12,34,37,38,40,43,44,47,49,52,54,57,59].

This study explored the relationship between WS and JB using 13 research samples. The test of homogeneity yielded a Q value of 123.7544, which was statistically significant (*p* < 0.001), thereby rejecting the null hypothesis of homogeneity and indicating substantial heterogeneity among the included studies. Accordingly, a random-effects model was employed. The I^2^ statistic was 89.99%, reflecting a high degree of heterogeneity. Based on the random-effects model, the estimated effect size between WS and JB was 0.5393, with a 95% confidence interval ranging from 0.4573 to 0.6213, reaching statistical significance. These findings demonstrate a positive association between WS and JB, and are illustrated in Figure 4. The leave-one-out sensitivity analysis, presented in Appendix C, Table A7, demonstrated that the exclusion of any single study did not materially affect the overall findings.

**Figure A3:**
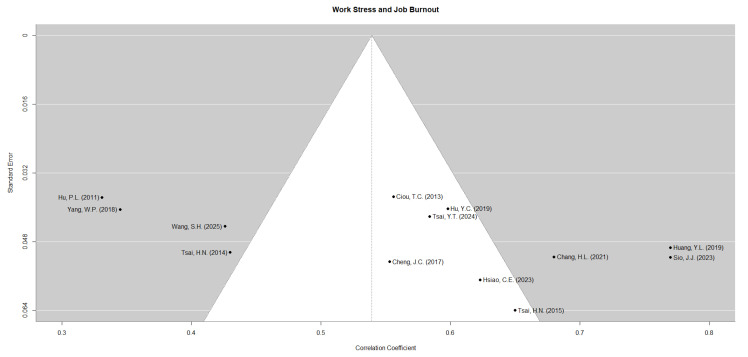
Funnel plot illustrating the effect size estimates from the meta-analysis of nurses’ work stress and job burnout in studies published between 2011 and 2025 (k = 13) [12,34,37,38,40,43,44,47,49,52,54,57,59].

To examine the potential for publication bias, the fail-safe N was calculated at 8860, indicating that this number of unpublished or missing studies with null results would be required to negate the observed association between WS and JB. This value greatly exceeds the commonly accepted threshold of 75, suggesting that the observed findings are robust and unlikely to be significantly affected by publication bias. A funnel plot depicting the distribution of effect size estimates across the included studies is provided in Appendix B, Figure A3. Moreover, Egger’s test for funnel plot asymmetry was not statistically significant (intercept = 0.0654, *p* = 0.0635), providing further support for the absence of publication bias.

### 3.3. Results of the Analysis Using Structural Equation Modeling

#### 3.3.1. The Relationships Among Work Stress, Job Burnout and Turnover Intention

**Figure 5:**
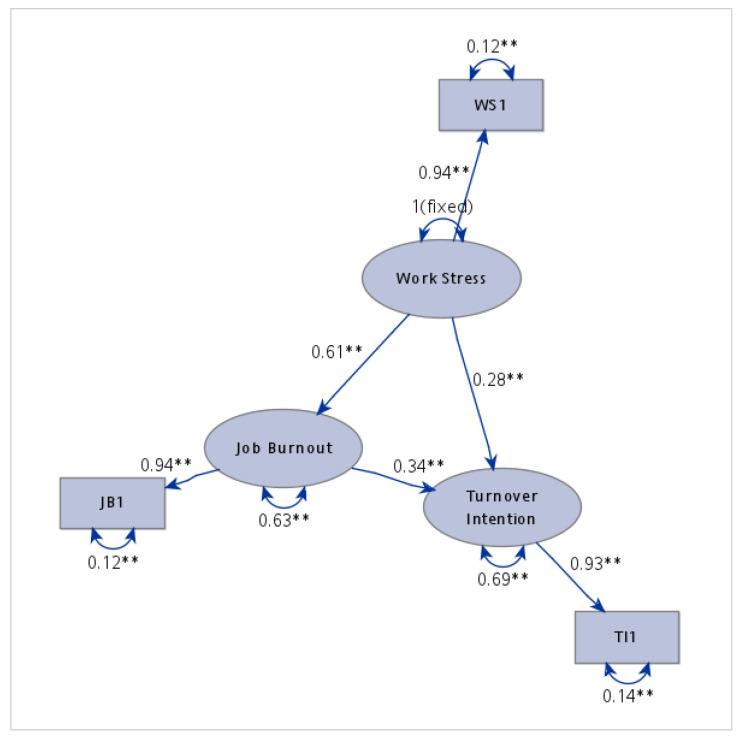
Structural model depicting standardized path coefficients for factors influencing nurses’ turnover intention. TI1 represents an observed indicator of the latent variable turnover intention; WS1 represents an observed indicator of the latent variable work stress; JB1 represents an observed indicator of the latent variable job burnout. The symbol "**" denotes statistical significance at the 0.01 level (*p* < 0.01).

Model parameters were estimated using the Maximum Likelihood (ML) method based on meta-analytic correlation coefficients. The results, shown in Figure 5, indicate a perfect fit (χ^2^ = 0, df = 0; RMSEA = 0). However, researchers applying MASEM to test causal models should interpret fit cautiously, as findings reflect consistency with the hypothesized structure rather than definitive causal evidence unless data originate solely from studies permitting causal inference [13].

The effect of WS on TI was statistically significant (path coefficient = 0.281, *p* = 0.002), thereby supporting Hypothesis 1 (H1). JB also exhibited a significant effect on TI (path coefficient = 0.342, *p* < 0.001), providing support for Hypothesis 2 (H2). Furthermore, WS had a significant positive effect on JB (path coefficient = 0.612, *p* < 0.001), supporting Hypothesis 3 (H3).

The results of the standardized bootstrap mediation analysis for the total effect of WS on TI are presented in Table 2. The total, direct, and indirect effects of WS on TI were examined. The findings indicated that the total effect was 0.490 (*p* < 0.001), comprising a direct effect of 0.281 (WS-TI; *p* = 0.002) and an indirect effect of 0.208 (WS-JB-TI; *p* < 0.001). These results suggest that WS influences TI both directly and indirectly through JB, thereby confirming the mediating role of JB (H4).

#### 3.3.2. Comparative Analysis of Turnover Intention Among Nursing Staff Across Diverse Healthcare Settings

Of the 28 studies included in this review, 5 were conducted in medical center settings [19,34,41,45,46], while 14 focused on non-medical center hospitals, including regional, district, and community hospitals [12,39,42,43,47,48,50,51,52,54,56,57,58,59]. The remaining 9 studies either encompassed multiple types of healthcare settings or did not explicitly report the study context. For further analysis, this study focused on the 19 studies that specifically examined medical centers and non-medical center hospitals. The findings are summarized in Table 3.

In the comparative analysis of different types of healthcare settings, significant differences were identified between medical centers and non-medical center hospitals across most pathways, with the exception of the relationship between JB and TI. Specifically, no statistically significant differences were found between nurses in medical centers and those in non-medical center hospitals regarding the effects of WS and JB on TI. However, the influence of WS on JB was significantly lower among nurses in medical centers compared to those in non-medical center hospitals (difference in estimates = −0.210, *p* = 0.037). These findings suggest that WS exerts a greater impact on JB among nurses employed in non-medical center hospitals in Taiwan.

#### 3.3.3. Comparative Analysis of Turnover Intention Among Nursing Staff Before and After the COVID-19 Pandemic

Among the 28 studies included in this analysis, 17 were conducted prior to the onset of the COVID-19 pandemic [11,12,19,35,36,37,38,39,40,41,42,43,44,45,46,58,59], while the remaining 11 were conducted during the post-pandemic period [47,48,49,50,51,52,53,54,55,56,57]. This study aims to examine potential differences in the structural relationships among WS, JB, and TI across the pre- and post-COVID-19 periods. The results of this comparative analysis are presented in Table 4.

The comparative analysis of studies conducted before and after the COVID-19 pandemic revealed no statistically significant differences in the relationships among the key variables. Specifically, the effect of WS on TI (difference in estimates = −0.108, *p* = 0.249) and the effect of JB on TI (difference in estimates = 0.268, *p* = 0.068) did not differ significantly between the two periods. Similarly, the relationship between WS and JB showed no significant variation (difference in estimates = −0.056, *p* = 0.700).

## 4. Discussion and Suggestion

This study investigates TI, WS, and JB among Taiwanese nurses by integrating these variables, which have traditionally been examined separately. Employing meta-analysis and structural equation modeling, the study explores the interrelationships among WS, JB, and TI, as well as their combined effects. It also compares these factors across different types of healthcare institutions and between the pre- and post-COVID-19 periods. The findings indicate that both WS and JB significantly contribute to increased TI, with JB serving as a mediator in the relationship between WS and TI. Moreover, the influence of these factors varies across healthcare settings.

The results indicate that WS significantly increases TI and is a strong contributor to JB. These findings are consistent with previous research [1], which has shown that prolonged exposure to high-stress work environments elevates nurses’ stress levels and TIs. Nurses face multiple sources of stress, including rotating shifts, patient care demands, and communication difficulties with patients’ families [1]. To address these challenges, healthcare organizations should implement stress management strategies, such as improving shift schedules and offering stress-relief programs [17]. Providing better psychological support can also help nurses cope, increase job satisfaction, and encourage them to stay in their roles.

The findings show that JB significantly affects nurses’ intention to leave their jobs. JB usually results from long-term emotional exhaustion and physical strain, especially in workplaces with limited resources or high stress levels [6]. It is an important factor that reduces nurses’ work efficiency and greatly increases their desire to quit [22,28]. To address this, healthcare organizations should improve resource support by reducing paperwork and creating teams to help nurses with non-clinical duties. Regular team-building initiatives and mental health programs have been shown to be effective in mitigating JB. Additionally, encouraging nurses to engage in professional development can boost their sense of achievement and strengthen their commitment to the organization.

Nurses in medical centers generally experience higher levels of work-related stress, primarily due to high patient turnover and the complexity of medical cases [15]. While previous studies have focused on differences in stress levels between medical centers and non-medical center hospitals [24], few have examined the specific effects of WS. The findings of this study indicate that the impact of WS on JB is greater in non-medical center hospitals than in medical centers. This may be attributed to the relatively stronger resource support available in medical centers, which appears to mitigate the effect of WS on JB. Based on these results, non-medical center hospitals are encouraged to strengthen human resource management by optimizing workload distribution and providing targeted psychological support to departments experiencing high stress. Furthermore, improvements in infrastructure, resource allocation, and access to professional training may help alleviate stress related to limited institutional resources.

A comparison of the relationships among TI, WS, and JB before and after the COVID-19 pandemic showed no statistically significant differences. The COVID-19 pandemic led to increased nurse attrition driven by heavier workloads, and the resulting workforce shortages further exacerbated WS [29] and JB [2] compared to pre-pandemic levels. However, the underlying relationships among WS, JB, and TI remained consistent across the two time periods.

### 4.1. Discussion

This study shows that nurses with higher levels of WS or JB are more likely to have stronger intentions to leave their jobs. In addition, increased stress causes more severe JB, which raises nurses’ intention to leave their profession. To reduce high turnover among clinical nursing staff, healthcare administrators must recognize and manage the stress nurses face. A thorough assessment of key areas such as human resources, work environment, organizational procedures, compensation, and nursing education should be conducted to identify the main causes of WSs and JB. Establishing systems for timely feedback and open, bidirectional communication can help alleviate WS and reduce the likelihood of nurse turnover.

This study found that the impact of WS on JB among nurses differs across healthcare setting types. The impact of WS on JB is significantly higher in non-medical center hospitals compared to medical centers. Medical centers face challenges such as higher patient turnover, more complex and severe cases, rapid changes in patient conditions, and the need for immediate medical interventions. Nurses in these centers must constantly update their knowledge and skills to handle urgent care. Additionally, greater involvement of patients’ families makes communication more difficult, which adds to stress. These factors can increase JB among nurses in medical centers. However, medical centers typically have more resources due to accreditation requirements, including better staffing, improved work environments, advanced equipment, professional training, standardized procedures, and stress management programs. Therefore, although work-related stress leads to JB in nurses at medical centers, its effect may be reduced by these supportive resources.

This study found no significant changes in the relationships between TI, WS, and JB before and after the COVID-19 pandemic. The effects of WS and JB on TI remained stable across these periods. However, many previous studies have reported that nurses experienced higher levels of WS and JB following the pandemic. Therefore, to reduce nurse turnover after the pandemic, it is important to address factors that have increased WS and JB, such as the risk of infection, family concerns about workplace safety, and the additional demands of infection prevention measures.

### 4.2. Suggestion

This study investigates the relationships among WS, JB, and TI among nurses in Taiwan, while also exploring how different healthcare settings and temporal changes before and after the COVID-19 pandemic may affect their work experiences. The results indicate that both WS and JB significantly contribute to TI among nurses. Moreover, JB mediates the relationship between WS and TI, suggesting that WS leads to increased JB, which in turn elevates nurses’ intention to leave their positions. The magnitude of these effects varies according to the type of healthcare setting and the temporal context before and after the COVID-19 pandemic. For example, the impact of WS on JB is more pronounced among nurses working in non-medical center hospitals compared to those in medical centers. Based on these findings, the following recommendations are proposed:

1. Development of stress management and support systems: Healthcare administrators should enhance stress management strategies for nurses by implementing targeted interventions that address specific sources of WS. These may include improving shift scheduling, promoting supportive workplace relationships, and offering regular mental health services and stress-relief programs [3].
2. Tailored policy development: Policies should be adapted to meet the specific needs of different types of healthcare institutions. In medical centers, strategies should focus on improving staffing levels and strengthening support for professional development. In non-medical center hospitals, priority should be given to enhancing the work environment and increasing resource investment to reduce stress caused by limited resources [26].
3. Long-term training and career development planning: The implementation of continuous education and career development programs can enhance nurses’ professional competencies and foster a greater sense of accomplishment, which may, in turn, mitigate TI associated with JB. [25].
4. Establishment of two-way communication channels: Facilitating opportunities for nurses to express their stress and needs can foster constructive communication between staff and the organization. Such open dialogue may enhance job satisfaction and contribute to reducing turnover risk [18].
5. WS and JB associated with COVID-19: Although the COVID-19 pandemic did not significantly modify the relationship between TI, WS, and JB, post-pandemic interventions aimed at reducing high turnover rates among nursing staff should prioritize targeting the distinct types of WS and JB that arose during the pandemic [2].

## 5. Conclusions

This study underscores the significant roles of WS and JB in influencing nurses’ TI. Employing structural equation modeling, it examines how these relationships vary across different healthcare settings and between the periods before and after the COVID-19 pandemic. The findings suggest that healthcare organizations and administrators should adopt targeted policies aimed at stress management, enhancement of the work environment, and support for career development. These measures are essential for mitigating nurse turnover and enhancing both the quality and efficiency of healthcare delivery.

This study focused exclusively on nurses in Taiwan. Due to cultural differences in workplace environments and work attitudes across countries, the findings may not be generalizable to other regions. However, the results offer valuable insights into nurses’ work attitudes within the context of Chinese culture.

## Figures and Tables

**Table 1:** Description of studies included in this review.

		n	%
Year of Publication	2011	2	7.14
	2012	1	3.57
	2013	2	7.14
	2014	2	7.14
	2015	1	3.57
	2016	1	3.57
	2017	3	10.71
	2018	1	3.57
	2019	4	14.29
	2020	0	0.00
	2021	1	3.57
	2022	1	3.57
	2023	4	14.29
	2024	4	14.29
	2025	1	3.57
Sample Size	1~99	2	7.14
	100~199	9	32.14
	200~299	7	25.00
	300~399	6	21.43
	400~499	3	10.71
	500~599	0	0.00
	600~699	0	0.00
	700~	1	3.57
Type of Sampled Institution	Medical Centers	5	17.86
	Regional Hospitals and Clinics	14	50.00
	Unspecified/Unknown	9	32.14
Research Time	Before COVID-19 Pandemic	17	60.71
	After COVID-19 Pandemic	11	39.29
Type of publication	Degree Thesis	24	85.71
	Journal Article	4	14.29
Total		28	100.00

**Table 2:** Testing total, direct, and indirect effects of work stress on turnover intention through standardized bootstrap mediation.

Path		Effect Size	SE	Bias-Corrected 95% CI			Accounts for the Total Effect Ratio
				Lower	Upper	*p*	
Indirect effect	WS-JB-TI	0.208	0.057	0.093	0.325	<0.001	42.53%
Direct effect	WS-TI	0.281	0.089	0.102	0.459	0.002	57.47%
Total effect		0.489	0.061	0.352	0.628	<0.001	100.00$

**Table 3:** An analysis of differences in standardized parameter estimates between nurses in medical centers and non-medical center hospitals.

Path	Medical Centers Path Coefficient	Non-Medical Center Hospitals Path Coefficient	Difference	*p*-Value
Work Stress -> Turnover Intention	0.278	0.066	0.213	0.071
Job Burnout -> Turnover Intention	0.493	0.549	−0.055	0.641
Work Stress -> Job Burnout	0.376	0.586	−0.210	0.037

**Table 4:** Comparative summary of standardized parameter estimates for nurses employed pre- and post-COVID-19 onset.

Path	Before COVID-19 Path Coefficient	After COVID-19 Path Coefficient	Difference	*p*-Value
Work Stress -> Turnover Intention	0.254	0.310	−0.108	0.249
Job Burnout -> Turnover Intention	0.443	0.175	0.268	0.068
Work Stress -> Job Burnout	0.575	0.683	−0.056	0.700

**Figure A1:**
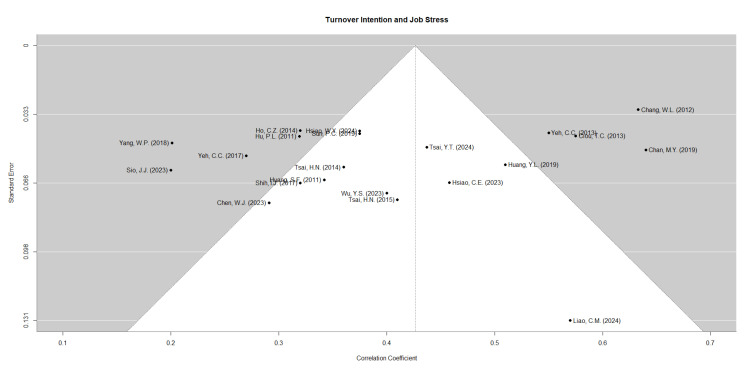
Funnel plot displaying the effect size estimates from the meta-analysis of work stress and nurses’ turnover intention in studies published between 2011 and 2025 (k = 21) [12,19,34,35,36,37,38,41,42,43,44,45,46,49,50,51,52,54,55,56,58].

## Data Availability

No new data were created or analyzed in this study. Data sharing is not applicable to this article.

## Abbreviations

The following abbreviations are used in this manuscript:

TI	Turnover Intention
WS	Work Stress
JB	Job Burnout
CFA	Confirmatory Factor Analysis
MASEM	Meta-Analytic Structural Equation Modeling
ML	Maximum Likelihood
SEM	Structural Equation Modeling

## Appendix A

**Table A1:** Appraisal Tool for Cross-Sectional Studies (AXIS)(1/4).

Study	Tsai, H.N. (2014) [12]	Huang, S.F. (2011) [19]	Hu, P.L. (2011) [34]	Chang, W.L. (2012) [35]	Yeh, C.C. (2013) [36]	Ciou, T.C. (2013) [37]	Tsai, H.N. (2015) [38]
Question							
**Introduction**							
1. Aims	Yes	Yes	Yes	Yes	Yes	Yes	Yes
**Methods**							
2. Study Design	Yes	Yes	Yes	Yes	Yes	Yes	Yes
3. Sample Size Justification	Yes	Yes	Yes	Yes	Yes	Yes	Yes
4. Target (Reference) Population	Yes	Yes	Yes	Yes	Yes	Yes	Yes
5. Sampling Frame	Yes	Yes	Yes	Yes	Yes	Yes	Yes
6. Sample Selection	Yes	Yes	Yes	Yes	Yes	Yes	Yes
7. Non-responders	Yes	Yes	Yes	Yes	Yes	Yes	Yes
8. Measurement Validity	Yes	Yes	Yes	Yes	Yes	Yes	Yes
9. Reliability	Yes	Yes	Yes	Yes	Yes	Yes	Yes
10. Statistics	Yes	Yes	Yes	Yes	Yes	Yes	Yes
11. Overall Methods	No	Yes	No	No	Yes	No	Yes
**Results**							
12. Basic Data	Yes	Yes	Yes	Yes	Yes	Yes	Yes
13. Response Rate	Yes	Yes	Yes	Yes	Yes	Yes	Yes
14. Non-responders Described	No	Yes	No	Yes	Yes	Yes	Yes
15. Internally Consistent Results	Yes	Yes	Yes	Yes	Yes	Yes	Yes
16. Comprehensive Description of Results	Yes	Yes	Yes	Yes	Yes	Yes	Yes
**Discussion**							
17. Justified Discussions and Conclusions	Yes	Yes	Yes	Yes	Yes	Yes	Yes
18. Limitations	Yes	Yes	Yes	Yes	Yes	Yes	Yes
**Other**							
19. Conflicts of Interest	No	No	No	No	No	No	No
20. Ethical Approval	No	No	No	No	No	No	No
Number of Yes	16	18	16	17	18	17	18

**Table A2:** Appraisal Tool for Cross-Sectional Studies (AXIS) (2/4).

Study	Huang, Y.P. (2016) [39]	Cheng, J.C. (2017) [40]	Shih, I.J. (2017) [41]	Yeh, C.C. (2017) [42]	Yang, W.P. (2018) [43]	Huang, Y.L. (2019) [44]	Sun, P.C. (2019) [45]
Question							
**Introduction**							
1. Aims	Yes	Yes	Yes	Yes	Yes	Yes	Yes
**Methods**							
2. Study Design	Yes	Yes	Yes	Yes	Yes	Yes	Yes
3. Sample Size Justification	Yes	Yes	Yes	Yes	Yes	Yes	Yes
4. Target (Reference) Population	Yes	Yes	Yes	Yes	Yes	Yes	Yes
5. Sampling Frame	Yes	Yes	Yes	Yes	Yes	Yes	Yes
6. Sample Selection	Yes	Yes	Yes	Yes	Yes	Yes	Yes
7. Non-responders	Yes	Yes	Yes	Yes	Yes	Yes	Yes
8. Measurement Validity	Yes	Yes	Yes	Yes	Yes	Yes	Yes
9. Reliability	Yes	Yes	Yes	Yes	Yes	Yes	Yes
10. Statistics	Yes	Yes	Yes	Yes	Yes	Yes	Yes
11. Overall Methods	No	Yes	Yes	Yes	Yes	Yes	Yes
**Results**							
12. Basic Data	Yes	Yes	Yes	Yes	Yes	Yes	Yes
13. Response Rate	Yes	Yes	Yes	Yes	Yes	Yes	Yes
14. Non-responders Described	No	Yes	Yes	No	Yes	Yes	Yes
15. Internally Consistent Results	Yes	Yes	Yes	Yes	Yes	Yes	Yes
16. Comprehensive Description of Results	Yes	Yes	Yes	Yes	Yes	Yes	Yes
**Discussion**							
17. Justified Discussions and Conclusions	Yes	Yes	Yes	Yes	Yes	Yes	Yes
18. Limitations	Yes	Yes	Yes	Yes	Yes	Yes	Yes
**Other**							
19. Conflicts of Interest	No	No	Yes	No	Yes	No	No
20. Ethical Approval	No	No	Yes	No	Yes	No	No
Number of Yes	17	18	20	17	20	18	18

**Table A3:** Appraisal Tool for Cross-Sectional Studies (AXIS) (3/4).

Study	Chan, M.Y. (2019) [46]	Chang, H.L. (2021) [47]	Lu, Y.H. (2022) [48]	Sio, J.J. (2023) [49]	Wu, Y.S. (2023) [50]	Chen, W.J. (2023) [51]	Hsiao, C.E. (2023) [52]
Question							
**Introduction**							
1. Aims	Yes	Yes	Yes	Yes	Yes	Yes	Yes
**Methods**							
2. Study Design	Yes	Yes	Yes	Yes	Yes	Yes	Yes
3. Sample Size Justification	Yes	Yes	Yes	Yes	Yes	Yes	Yes
4. Target (Reference) Population	Yes	Yes	Yes	Yes	Yes	Yes	Yes
5. Sampling Frame	Yes	Yes	Yes	Yes	Yes	Yes	Yes
6. Sample Selection	Yes	Yes	Yes	Yes	Yes	Yes	Yes
7. Non-responders	Yes	Yes	Yes	Yes	Yes	Yes	Yes
8. Measurement Validity	Yes	Yes	Yes	Yes	Yes	Yes	Yes
9. Reliability	Yes	Yes	Yes	Yes	Yes	Yes	Yes
10. Statistics	Yes	Yes	Yes	Yes	Yes	Yes	Yes
11. Overall Methods	Yes	Yes	Yes	Yes	Yes	Yes	Yes
**Results**							
12. Basic Data	Yes	Yes	Yes	Yes	Yes	Yes	Yes
13. Response Rate	Yes	Yes	Yes	Yes	Yes	Yes	Yes
14. Non-responders Described	Yes	Yes	No	Yes	Yes	Yes	No
15. Internally Consistent Results	Yes	Yes	Yes	Yes	Yes	No	Yes
16. Comprehensive Description of Results	Yes	Yes	Yes	Yes	Yes	Yes	Yes
**Discussion**							
17. Justified Discussions and Conclusions	Yes	Yes	Yes	Yes	Yes	Yes	Yes
18. Limitations	Yes	Yes	Yes	Yes	Yes	Yes	Yes
**Other**							
19. Conflicts of Interest	Yes	Yes	Yes	No	Yes	No	Yes
20. Ethical Approval	Yes	Yes	Yes	No	Yes	No	Yes
Number of Yes	20	20	19	18	20	17	19

**Table A4:** Appraisal Tool for Cross-Sectional Studies (AXIS) (4/4).

Study	Chen, Y.L. (2024) [53]	Tsai, Y.T. (2024) [54]	Hsiao, W.Y. (2024) [55]	Liao, C.M. (2024) [56]	Wang, S.H. (2025) [57]	Ho, C.Z. (2014) [58]	Hu, Y.C. (2019) [59]
Question							
**Introduction**							
1. Aims	Yes	Yes	Yes	Yes	Yes	Yes	Yes
**Methods**							
2. Study Design	Yes	Yes	Yes	Yes	Yes	Yes	Yes
3. Sample Size Justification	Yes	Yes	Yes	Yes	Yes	Yes	Yes
4. Target (Reference) Population	Yes	Yes	Yes	Yes	Yes	Yes	Yes
5. Sampling Frame	Yes	Yes	Yes	Yes	Yes	Yes	Yes
6. Sample Selection	Yes	Yes	Yes	Yes	Yes	Yes	Yes
7. Non-responders	Yes	Yes	Yes	Yes	Yes	Yes	Yes
8. Measurement Validity	Yes	Yes	Yes	Yes	Yes	Yes	Yes
9. Reliability	Yes	Yes	Yes	Yes	Yes	Yes	Yes
10. Statistics	Yes	Yes	Yes	Yes	Yes	Yes	Yes
11. Overall Methods	Yes	Yes	Yes	Yes	Yes	Yes	No
**Results**							
12. Basic Data	Yes	Yes	Yes	Yes	Yes	Yes	Yes
13. Response Rate	Yes	Yes	Yes	Yes	Yes	Yes	Yes
14. Non-responders Described	No	Yes	No	Yes	Yes	Yes	No
15. Internally Consistent Results	Yes	Yes	Yes	Yes	Yes	Yes	Yes
16. Comprehensive Description of Results	Yes	Yes	Yes	Yes	Yes	Yes	Yes
**Discussion**							
17. Justified Discussions and Conclusions	Yes	Yes	Yes	Yes	Yes	Yes	Yes
18. Limitations	Yes	Yes	Yes	Yes	Yes	Yes	Yes
**Other**							
19. Conflicts of Interest	No	Yes	No	Yes	Yes	No	No
20. Ethical Approval	No	Yes	No	Yes	Yes	No	No
Number of Yes	17	20	17	20	20	18	16

## Appendix C

**Table A5:** Leave-one-out sensitivity analyses were conducted to examine the relationship between work stress and turnover intention.

Removed Study	Estimate	SE	z-Value	*p*-Value	95%CI.lb	95%CI.ub	Q	tau2	I2	H2
Hu, P.L. (2011) [34]	0.4336	0.0337	12.8842	<0.001	0.3677	0.4996	154.1945	0.0153	85.4965	6.8949
Huang, S.F. (2011) [19]	0.4289	0.0335	12.8125	<0.001	0.3633	0.4945	158.9783	0.0154	86.0848	7.1864
Chang, W.L. (2012) [35]	0.3962	0.0295	13.4514	<0.001	0.3384	0.4539	108.3698	0.0122	81.5565	5.4220
Ciou, T.C. (2013) [37]	0.4161	0.0324	12.8313	<0.001	0.3526	0.4797	148.0434	0.0140	84.4146	6.4163
Yeh, C.C. (2013) [36]	0.4172	0.0329	12.6839	<0.001	0.3527	0.4816	151.2573	0.0145	84.7663	6.5644
Ho, C.Z. (2014) [58]	0.4347	0.0337	12.9175	<0.001	0.3687	0.5006	153.3031	0.0153	85.3829	6.8413
Tsai, H.N. (2014) [12]	0.4288	0.0337	12.7250	<0.001	0.3627	0.4948	159.4058	0.0156	86.0924	7.1903
Tsai, H.N. (2015) [38]	0.4267	0.0335	12.7463	<0.001	0.3611	0.4923	160.7142	0.0156	86.2905	7.2942
Shih, I.J. (2017) [41]	0.4294	0.0333	12.8958	<0.001	0.3641	0.4947	158.0510	0.0153	85.9777	7.1315
Yeh, C.C. (2017) [42]	0.4334	0.0329	13.1601	<0.001	0.3689	0.4980	151.4830	0.0147	85.2688	6.7883
Yang, W.P. (2018) [43]	0.4395	0.0316	13.9260	<0.001	0.3777	0.5014	135.8616	0.0132	83.7396	6.1499
Sun, P.C. (2019) [45]	0.4301	0.0341	12.6270	<0.001	0.3633	0.4968	159.1602	0.0157	85.7750	7.0299
Huang, Y.L. (2019) [44]	0.4231	0.0333	12.7015	<0.001	0.3578	0.4884	158.5114	0.0151	85.7405	7.0128
Chan, M.Y. (2019) [46]	0.4156	0.0310	13.4218	<0.001	0.3549	0.4762	141.4391	0.0127	83.2901	5.9845
Wu, Y.S. (2023) [50]	0.4270	0.0335	12.7336	<0.001	0.3613	0.4927	160.6211	0.0156	86.2760	7.2865
Chen, W.J. (2023) [51]	0.4293	0.0329	13.0367	<0.001	0.3648	0.4938	157.4351	0.0150	85.8732	7.0788
Sio, J.J. (2023) [49]	0.4343	0.0317	13.7143	<0.001	0.3722	0.4964	145.7067	0.0135	84.3686	6.3974
Hsiao, C.E. (2023) [52]	0.4254	0.0335	12.6891	<0.001	0.3597	0.4911	160.5247	0.0155	86.1583	7.2246
Tsai, Y.T. (2024) [54]	0.4258	0.0340	12.5275	<0.001	0.3592	0.4924	160.7149	0.0157	85.9773	7.1313
Hsiao, W.Y. (2024) [55]	0.4303	0.0341	12.6370	<0.001	0.3636	0.4971	159.0461	0.0157	85.7217	7.0036
Liao, C.M. (2024) [56]	0.4253	0.0324	13.1114	<0.001	0.3617	0.4889	159.5560	0.0149	85.9485	7.1167

**Table A6:** Leave-one-out sensitivity analyses were conducted to assess the relationship between job burnout and turnover intention.

Removed Study	Estimate	SE	z-Value	*p*-Value	95%CI.lb	95%CI.ub	Q	tau2	I2	H2
Hu, P.L. (2011) [34]	0.4353	0.0766	5.6819	<0.001	0.2851	0.5854	169.1900	0.0518	94.7462	19.0339
Ciou, T.C. (2013) [37]	0.4254	0.0751	5.6640	<0.001	0.2782	0.5725	159.7679	0.0497	94.5324	18.2894
Tsai, H.N. (2014) [12]	0.4603	0.0747	6.1630	<0.001	0.3139	0.6067	159.8326	0.0493	94.8061	19.2535
Tsai, H.N. (2015) [38]	0.4655	0.0628	7.4059	<0.001	0.3423	0.5886	134.2705	0.0352	93.0686	14.4271
Huang, Y.P. (2016) [39]	0.4211	0.0702	6.0016	<0.001	0.2836	0.5587	142.7811	0.0429	93.9957	16.6546
Yang, W.P. (2018) [43]	0.4344	0.0765	5.6782	<0.001	0.2844	0.5843	167.8908	0.0513	94.7853	19.1765
Huang, Y.L. (2019) [44]	0.4305	0.0735	5.8600	<0.001	0.2865	0.5744	159.4206	0.0475	94.6034	18.5302
Lu, Y.H. (2022) [48]	0.4446	0.0734	6.0541	<0.001	0.3007	0.5886	172.2648	0.0511	95.2845	21.2065
Sio, J.J. (2023) [49]	0.4738	0.0651	7.2740	<0.001	0.3461	0.6015	122.9635	0.0370	93.2291	14.7692
Hsiao, C.E. (2023) [52]	0.4453	0.0765	5.8182	<0.001	0.2953	0.5952	172.5814	0.0525	95.1888	20.7849
Chen, Y.L. (2024) [53]	0.4726	0.0751	6.2921	<0.001	0.3254	0.6198	152.5209	0.0503	94.5244	18.2630
Tsai, Y.T. (2024) [54]	0.4380	0.0769	5.6938	<0.001	0.2872	0.5888	170.2241	0.0518	94.8839	19.5462

**Table A7:** Leave-one-out sensitivity analyses were conducted to evaluate the relationship between work stress and job burnout.

Removed Study	Estimate	SE	z-Value	*p*-Value	95%CI.lb	95%CI.ub	Q	tau2	I2	H2
Hu, P.L. (2011) [34]	0.5660	0.0397	14.2634	<0.001	0.4883	0.6438	89.0229	0.0154	87.5247	8.0158
Ciou, T.C. (2013) [37]	0.5371	0.0455	11.7993	<0.001	0.4479	0.6264	123.5258	0.0209	90.4921	10.5175
Tsai, H.N. (2014) [12]	0.5467	0.0439	12.4487	<0.001	0.4606	0.6328	118.7145	0.0192	90.2655	10.2728
Tsai, H.N. (2015) [38]	0.5347	0.0442	12.0924	<0.001	0.4481	0.6214	120.6033	0.0200	90.8194	10.8925
Cheng, J.C. (2017) [40]	0.5385	0.0454	11.8593	<0.001	0.4495	0.6275	123.6809	0.0208	90.9639	11.0667
Yang, W.P. (2018) [43]	0.5606	0.0407	13.7710	<0.001	0.4808	0.6404	97.9513	0.0161	88.2179	8.4875
Hu, Y.C. (2019) [59]	0.5328	0.0455	11.7009	<0.001	0.4436	0.6221	121.3733	0.0207	90.5577	10.5906
Huang, Y.L. (2019) [44]	0.5229	0.0410	12.7578	<0.001	0.4426	0.6032	100.1588	0.0165	88.7889	8.9198
Chang, H.L. (2021) [47]	0.5302	0.0440	12.0405	<0.001	0.4439	0.6165	115.7379	0.0194	90.3557	10.3688
Sio, J.J. (2023) [49]	0.5244	0.0410	12.7850	<0.001	0.4440	0.6048	102.3325	0.0166	88.9074	9.0150
Hsiao, C.E. (2023) [52]	0.5349	0.0448	11.9277	<0.001	0.4470	0.6228	121.4471	0.0203	90.8778	10.9623
Tsai, Y.T. (2024) [54]	0.5348	0.0457	11.7134	<0.001	0.4453	0.6243	122.4991	0.0208	90.6674	10.7151
Wang, S.H. (2025) [57]	0.5494	0.0439	12.5110	<0.001	0.4634	0.6355	116.6191	0.0191	90.0134	10.0135
